# Development of a Reference Method and Materials for Quantitative Measurement of UV-Induced DNA Damage in Mammalian Cells: Comparison of Comet Assay and Cell Viability

**DOI:** 10.1155/2022/9188636

**Published:** 2022-09-17

**Authors:** Donald H. Atha, Alessandro Tona, Vytas Reipa

**Affiliations:** National Institute of Standards and Technology, Biosystems and Biomaterials Division, Material Measurement Laboratory, Gaithersburg, MD 20899, USA

## Abstract

Application of DNA damage diagnostic tests is rapidly growing, in particular for ovarian, prostate, and skin cancers; environmental monitoring; chronic and degenerative diseases; and male infertility. Such tests suffer from significant variability among different laboratories due the lack of standardization, experimental validation, and differences in data interpretation. Reference methods and materials for quantitative measurement of UVA-induced DNA damage in mammalian cells are frequently needed. In this study, we examined the use of the single-cell gel electrophoresis (comet) assay to assess the UVA-induced DNA damage in surface-attached Chinese hamster ovary (CHO) cells treated with a photosensitizer as a candidate cellular oxidative damage reference material. We found that the comet images became diffused and the viability of the cells decreased substantially (>20%) as the UVA dose and benzo [a] pyrene (BaP) concentration exceeded 6.3 J/cm^2^ and 10^−6^ mol/L BaP. Maintaining the conditions of exposure within this range can improve DNA damage measurement fidelity, particularly if used as a quantitative reference method and to produce materials considered as an *in vitro* standard for the comet assay.

## 1. Introduction

DNA is susceptible to damage from exogenous sources, in particular such as chemical carcinogens, ionizing and UV radiation [1]. Fortunately, intricate cellular mechanisms have evolved in mammalian cells to repair this damage to reduce permanent genetic alteration. Defects in DNA damage response pathways culminate in genomic instability in cancers, which is associated with aggressive disease and poor patient outcome. Any condition leading to high levels of DNA damage will result in replication stress, which could be a feature of precancerous and cancerous cells. However, when the damage exceeds certain levels or is of a particular type (e.g., double-strand breaks), the repair is not fully compensatory and the cell resorts to apoptosis or programmed cell death to prevent defective genetic information propagation in normal cell division. If the damage to genomic DNA includes proto-oncogenes and tumor suppressor genes such as RAS, MYC, and p53, the repair mechanism eventually leads to cancerous cells [2, 3]. Since cancer and numerous other diseases have been implicated as a result of such damage, it is critically important to reliably quantify DNA damage in order to compare data across research and diagnostic labs [4–6].

One of the most ubiquitous causes of exogenous DNA damage is UVA radiation, present in the solar spectrum (320 nm < *λ* < 400 nm) [7]. Mechanisms of DNA damage from UVA radiation have been studied extensively in the context of UV phototoxicity [8–10]. The effect of polycyclic aromatic hydrocarbons (PAH), such as benzo [a] pyrene (BaP), serving as photosensitizers during UVA exposure to plants and animals was shown to occur via triplet-state-induced singlet oxygen (^1^O_2_), or type II phototoxicity [11, 12]. Meanwhile, exposure to BaP in the dark [13–15] does not induce the genotoxic response. For *in vitro* experiments, this method of induced DNA damage to cultured cells is particularly advantageous in that it can be used to reproducibly cause extensive DNA double-strand breaks at relatively low levels of BaP [13, 15].

Multiple assays exist for the analysis of damage to genomic DNA [16, 17]. Some of these assays involve the measurement of structural changes to the DNA bases to form lesions that can be detected by chromatographic methods [13, 18, 19]. Other methods include measurement of DNA strand breakage in whole cells using the single-cell gel electrophoresis (comet assay) [20], the micronucleus assay [21], and the measurement of DNA fragmentation in isolated DNA using capillary or gel electrophoresis [22–24]. Application of DNA damage diagnostic tests is rapidly growing, in particular for ovarian, prostate, and skin cancers; environmental monitoring; chronic and degenerative diseases; and male infertility [25, 26]. Unfortunately, such tests suffer from significant variability among different laboratories [27] due to the lack of standardization, experimental validation, and differences in data interpretation [28]. Reference materials that are specifically designed for each of these assays may reduce this variability, facilitate standardization, and enable result comparison between laboratories [29].

In this report, we focus on a potential reference method for the testing of UV-induced DNA damage and generation of a candidate reference material, which utilizes viable mammalian cells, as required for many current assay systems. Such a reference method and material can also be applicable to evaluate the effectiveness of photosensitizers as well as antioxidants used for diagnostic purposes [30].

The alkaline comet assay is a sensitive method that detects both single- and double-strand breaks in individual nonproliferating cells in a wide variety of cell types [28, 31–35]. The method is based on the migration of cleaved DNA out of the nuclei in an electric field, with the intact DNA remaining within the nucleoid. Microscopic imaging the comet tail and nucleoid allows for relative assessment of the percentage of damaged DNA. The neutral comet assay is predominantly sensitive to double-stand breaks. The alkaline comet assay used in this study includes both single- and double-strand breaks. However, single- and double-strand breaks produce different electrophoretic patterns in the resulting imaged comet profiles. This leads to inaccuracies in comparing data when the cells differ in the type of exposure as well as the condition of the cells [36, 37]. For this reason, rather than alternative methods such as the olive tail moment, we have chosen to measure the percentage DNA in the tail using the alkaline comet assay to minimize these effects and more directly assess the total percentage of DNA single- and double-strand breaks [38].

We have assessed the cytotoxic and genotoxic effects of BaP and UVA exposure on surface-attached CHO cells as a candidate reference material for DNA damage. The cells were analyzed by comet and viability assays in parallel under a range of UVA exposures and BaP doses. Cell viability was evaluated using MTS assay that offers an efficient screening technique that is based on the reduction of the MTS tetrazolium compound to generate a colored formazan dye produced by NAD(P)H-dependent dehydrogenase enzymes in metabolically active mammalian cells [39]. The optical density of the culture media, measured at 490 nm, follows the live cell count and is compared to the extent of DNA strand breaks assessed by the average percentage of DNA measured in the tails of the comet assay images.

## 2. Materials and Methods

### 2.1. Cell Culture

Stock cultures of Chinese hamster ovary CHO K1 cells (ATCC, Manassas, VA, USA) were grown for 24 h at 37°C, 5% CO_2_, and 95% rel. humidity in Iscove's modified Dulbecco's modified medium (IMDM) (Gibco, Carlsbad, CA), 10% (*v*/*v*) fetal bovine serum (FBS) (Gibco), and 1% (*v*/*v*) penicillin-streptomycin (100 units/mL and 100 *μ*g/mL). Under these conditions, cells were grown to confluence on multiwell tissue culture-treated flat bottom clear polystyrene plates (Corning Life Sciences, Corning, NY) and treated by UVA/BaP as described as follows.

### 2.2. Cell Exposure to UVA and BaP

On day one, a black polystyrene 96-well plate was seeded with 5000 cells/100 *μ*L per well in IMDM media and incubated for 48 h. On day three, the supernatant media was decanted, the attached cells were rinsed with PBS (37°C), and four replicates of a series dilution of BaP at five concentrations (10^−7^ to 10^−4^) mol/L in media including a negative control (media only) were added to the attached cells. Following a 1 h incubation (37°C, 5% CO_2_, and 95% rel. humidity), the cells were given three different doses of UVA using a Fisher Scientific DNA crosslinker, equipped with 6f3t4-blb lamps operated at *λ* = 365 nm. The light intensity was measured at 3.5 mW/cm^2^ using a Black-ray (Upland, CA) model j-221 UV meter. First, two rows of the 96-well plate were exposed to UVA for 10 min. (UVA dose *P* = 2.1 J/cm^2^). During this initial exposure, the remaining six rows were shielded using a thick black paper. Next, the shield was moved to expose two more rows and again the plate exposed for another 10 min to UVA. Then, the shield was removed and two more rows were exposed for 10 min. Thus, the wells with five concentrations of BaP, control, and (10^−7^ to 10^−4^) mol/L were exposed to three doses of UVA, totaling 10 min., 20 min., and 30 min. (2.1 J/cm^2^, 4.2 J/cm^2^, and 6.3 J/cm^2^), respectively, with two rows left in the dark.

### 2.3. Viability Assay

The cytotoxic effect of BaP and UVA exposure was tested on CHO K1 cells using the MTS (3-(4,5-dimethylthiazol-2-yl)-5-(3-carboxymethoxyphenyl)-2-(4-sulfophenyl)-2H-tetrazolium\PMS-phenazine methosulfate cell viability assay CellTiter 96 AQueous One Solution Cell Proliferation Assay (MTS) (Promega, Madison, WI, USA).

The measurement procedure was adopted from ISO 19007:2018 “nanotechnologies—*in vitro* MTS assay for measuring the cytotoxic effect of nanoparticles” [40]. Following UVA exposure, the 96-well plate was rinsed with PBS and filled with 100 *μ*L of the MTS reagent in IMDM media. After 1 h incubation at 37°C, 5% CO_2_, and 95% relative humidity, the absorbance was recorded at 490 nm using the Biotek Model B2 multiplate reader. The average absorbance background level for BaP in culture medium was subtracted from each well. The resulting absorbance from each well was normalized to the absorbance value in the no-BaP (media only) wells. In addition, cells treated with 100 *μ*g/mL cisplatin (cisPt) as a positive control provided a reference to adjust for zero viability. That way the background-subtracted and normalized values represent the fraction of cells that retained viability after BaP/UVA dose treatment.

### 2.4. Comet Assay

Cells were seeded, grown, and treated with BaP and UVA as described above for the MTS assay except in six-well (1 mL/well) and twelve-well (2 mL/well) plates (at an equivalent cell density of 5 × 10^4^ cell/mL) in order to obtain adequate cell numbers for comet analysis. After the 48 h incubation, the confluent cells were treated at increasing BaP concentrations (10^−7^ to 10^−5^) mol/L and UVA exposure (2.1 J/cm^2^ and 6.3 J/cm^2^) by shielding of the plate rows as described above. A row with control cells was blocked from UVA exposure as described above. Within about 5 min. following UVA exposure, the control and treated cells were harvested by trypsin EDTA (2.5 mg/mL) and measured by alkaline comet assay as described previously [41–43] and again as follows. Low-melting point agarose, 300 *μ*L (Trevigen Inc., MD, USA, cat. no. 4250-050-02), was heated to 37°C and combined with 30 *μ*L (ratio 1 : 10 volume fraction) of an approximately 2 × 10^5^ cells per mL suspension of thoroughly mixed cells as collected above. Each well of a 20-well CometSlide (Trevigen Inc., MD, USA, cat. no. 4252-200-01) was filled with 30 *μ*L of a thoroughly mixed cell/agarose suspension. Slides were set up using five wells at each treatment concentration. The slides were placed in a 4°C refrigerator in the dark for 15 min to solidify. The slides were then immersed in 50 mL of prechilled lysis solution (3.2% *w*/*w* glycine, N, N′-1,2 ethanediylbis[N-(carboxymethyl)]-, 1% *w*/*w* n-dodecylsarcosine, 1% poly(oxy-1,2-ethanedyl), alpha-[4-1,1,3,3-tetramethylbutylphenyl]-omega-hydoxy, Trevigen Inc., cat. no. 4250-010-01) and left at 4°C for 30 min to facilitate cell membrane and histone removal. After draining excess liquid, the slides were transferred to 50 mL of freshly prepared (same day) alkaline solution (200 mmol/L NaOH, 1 mmol/L ethylene diamine tetraacetate (EDTA), and pH > 13) and incubated at room temperature in the dark for 20 min to denature and unwind DNA. After the unwinding step, electrophoresis was performed at 4°C in the Trevigen CometAssay ES tank (including spacer) filled with alkaline solution (200 mmol/L NaOH, 1 mmol/L EDTA) at 21 V (1 V/cm) for 30 min. The slides were then rinsed with distilled water and fixed 5 min. in 70% ethanol. The slides were dried and stained 5 min. at 4°C with SYBR Green I (Trevigen Inc., cat. no. 4250-050-05) diluted 1 : 10 000 in TE buffer pH 7.5 (10 mmol/L Tris, 1 mmol/L EDTA), drained to remove excess staining solution, and thoroughly dried at room temperature in the dark. With a convenient excitation and emission spectrum, the SYBR Green I dye binds to double stranded DNA to produce about a 1000-fold increase in fluorescence compared to free dye and only about a ten-fold lower binding to single stranded DNA. The use of alternative dyes may lead to reduced sensitivity and quality of the assay.

### 2.5. Microscopic Image Analysis of Comet Slides

Slides were visualized by epifluorescence microscopy using an Olympus Systems Model BH-2 microscope (Center Valley, PA), equipped with the appropriate optical filter set for SYBR Green I (460 nm excitation and 560 nm emission wavelengths, Chroma, 49002 ET GFP). A LUDL MAC 6000 automated stage and a Photometrics CoolSNAP HQ2 monochrome CCD camera (Hawthorne, NY) were controlled using Nikon Elements BR software (ver. 4.20, Nikon Inc., Tucson, AZ). Integrated intensities and percent DNA in the tail were determined using ImageJ (ver. 1.47v, NIH) and CometScore Pro (ver. 1.01.44, Tritek Corp., Herndon, VA) software utilizing the following equations:

(1) $$\begin{array}{c} \text{Total head intensity }I_{h}=\sum I_{h\left(x,y\right)},\\ \text{Total tail intensity }I_{t}=\sum I_{t\left(x,y\right)},\\ \%\text{DNA in tail}=100\frac{I_{t}}{\left(I_{h}+I_{t}\right)}, \end{array}$$

where *I*_*h*(*x*, *y*)_ and *I*_*t*(*x*, *y*)_ are the individual pixel intensities within the head and tail regions of the comet image. Although other methods can be used for the analysis of comets, such as the olive tail moment (OTM) [44], the % DNA in the tail was chosen here since it is appropriate for regulatory and interlaboratory comparison studies with minimal variability [45]. In addition, % DNA in the tail could be simply compared to the % loss in cell viability. CometScore Pro is commercially available software which has been specifically developed to automate comet image analysis. As described previously [41–43], we used our automated microscope system, controlled by Nikon Elements software, in combination with the CometScore Pro software to quantify DNA damage as % DNA in the tail in our cultured CHO cells with and without UVA and BaP treatment. Approximately 50 to 100 cells were counted in slide well replicates at each treatment level.

## 3. Results and Discussion

**Figure 1:**
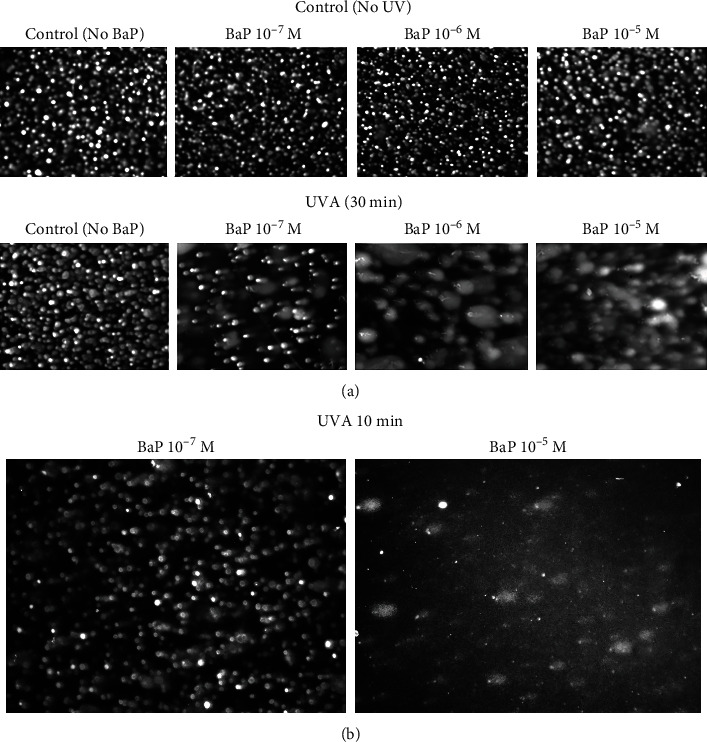
Comet assay sample images under various UVA and BaP treatment conditions. BaP concentration (10^−7^ to 10^−5^) mol/L. UVA exposure times were at an intensity of 3.5 mW/cm^2^. (a) 30 min dose = 6.3 J/cm^2^. (b) 10 min dose = 2.1 J/cm^2^. M: mol/L (see Materials and Methods).

Figure 1 shows sample comet assay images under a range of treatment conditions.  Figure 1(a) shows a clear decline in the cell density, and an increase in comet tails at 30 min UVA as the BaP concentration exceeds 10^−7^ mol/L BaP. The diminishing cell density was expected due to the loss of the cell structure and adhesion to the plate surface as fewer cells remain viable. In addition, starting at 10^−6^ mol/L BaP, large comets with very small nuclei, caused by highly fractured DNA, sometimes referred to as clouds or hedgehog comets, appear in large proportion [46]. At the highest UVA treatment levels, the measurement of the percentage DNA in the tail was hampered not only by the reduction in the number of available comets but by the amount of DNA in the large overlapping tail portions compared to the minute amount of nuclear material of each comet. In studies where differences in the type of DNA damage (i.e., proportion of crosslinks and single- and double-strand breaks) need to be detected, the use of the olive tail moment may be preferable, which is sensitive to the distribution of DNA in the comet tail [44, 45]. Occasional fragments of stained nuclear material and cellular debris from broken cells were also observed. As shown in Figure 1(b), UVA exposure for 10 min (2.1 J/cm^2^) at the highest BaP concentration did not result in a large population of overlapping comets and analysis was not problematic. Despite complications at the highest UVA treatment level, the average percentage DNA in the tail was determined for an adequate number of comets at each treatment level.

**Figure 2:**
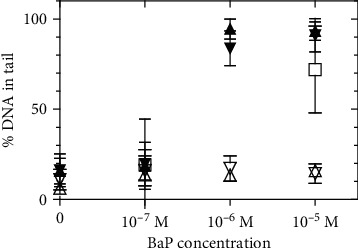
Comet assay results plotted as percentage DNA in the tail. M: mol/L. Open triangles: control no UV exposure. Filled triangles: UVA exposure of 30 min at an intensity of 3.5 mW/cm^2^ (6.3 J/cm^2^). Data from 2 separate replicate experiments is included. Open squares: UVA 10 min exposure (2.1 J/cm^2^). M: mol/L. Results were calculated and plotted as the mean and standard deviation (see Materials and Methods).

Figure 2 shows a plot of the percentage DNA in tail acquired from multiple images at each of the treatment levels shown in Figure 1. The pronounced increase in percentage DNA in the tail shown in Figure 2 demonstrates a sharp increase in strand breaks as the level of UVA exposure increases beyond 10 min (2.1 J/cm^2^) and the BaP concentration exceeds 10^−7^ mol/L. Figure 2 also indicates a higher standard error in the measurement of the percentage DNA in the tail that occurs at 30 min (6.3 J/cm^2^) UVA exposure at 10^−7^ mol/L BaP and at 10 min (2.1 J/cm^2^) exposure at 10^−5^ mol/L, which is due to the increased heterogeneity of these populations of comets.

**Figure 3:**
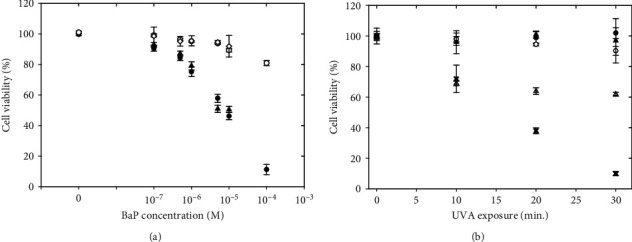
Loss of cell viability as the level of concurrent UVA and BaP exposure increases. (a) Effect of BaP concentration (10^−7^ to 10^−4^) mol/L with 30 min UVA exposure (filled symbols) and without (open symbols). Data from 2 separate replicate experiments is included. (b) Effect of increasing UVA exposure (10 min = 2.1 J/cm^2^, 20 min = 4.2 J/cm^2^, and 30 min = 6.3 J/cm^2^) and increasing BaP concentration: media only (▲); 10^−7^ M (●); 10^–6^ M (o); 10^−5^ M (*Δ*); 10^−4^ M (x). The data point at 10^−4^ M BaP, 10 min. UVA exposure was obtained from a different but equivalent set of experiments. UVA exposure times were at an intensity of 3.5 mW/cm^2^. M: mol/L. Results were calculated and plotted as the mean and standard deviation (see Materials and Methods).

Figures 3(a) and 3(b) show the decline in cell viability that occurs as the level of concurrent UVA and BAP exposure increases beyond 10 min (2.1 J/cm^2^) and 10^−7^ mol/L BaP.  Figure 3(a) emphasizes the differences observed with increased BaP concentration at high UVA dose (30 min, 6.3 J/cm^2^), compared to controls in the absence of UVA light, which follows the rise in strand breaks as shown in Figure 2. The data presented in Figure 3(b) demonstrates the additive effect of increasing the UVA dose and BaP concentration. We have also tested the protective effect of reduced glutathione (GSH) and sodium azide against cell damage by UVA/BaP (data not shown). Addition of GSH at 10 mmol/L did not restore cell viability, whereas sodium azide at 100 mmol/L prevented cell viability loss up to 20% with UVA exposures from 10 min to 30 min UVA and 10^−5^ mol/L BaP. Such action confirms the type II phototoxicity mechanism of DNA damage by photoexcited BaP since sodium azide is known to quench photo-induced singlet oxygen [47].

High levels of UVA and BaP may have a particularly damaging effect on the cell structure and function, as reflected by the large comets and loss of viability observed in this study. Our previous investigation of DNA damage caused by etoposide, bleomycin, and ethyl methanesulfonate (EMS) was performed under conditions where the cell viability was maintained at 96% to 98% [43]. The comet assay recorded DNA damage of as much as 82% when cells were exposed to 1600 *μ*g/mL EMS under these conditions. In the current study, with a UVA dose of 6.3 J/cm^2^ and BaP concentration of 10^−6^ mol/L, the comet assay data shows nearly complete DNA fragmentation but the viability was lower (≈80%). However, most of the treated cells were not apoptotic and still able to undergo DNA repair and survive. This is consistent with the findings of Lorenzo et al. [46] that showed recovery of cells that produced hedgehog comets after exposure to hydrogen peroxide. A substantial reduction in viability (≈40%) arises when the BaP concentration reaches 10^−5^ mol/L at a 30 min UVA dose of 6.3 J/cm^2^. Therefore, maintaining the cell exposure conditions within 10^−6^ mol/L BaP would improve the assay quality when viable cells are required, such as the comet assay.

The comet assay is a popular genotoxicity assay which has the advantage of directly examining DNA strand breakage in individual cells. Although subject to substantial variability and difficult to quantify and automate, particularly without standard protocols and reference materials, the method is relatively simple and efficient and uses equipment commonly found in biological research and clinical diagnostic laboratories [28, 33, 34]. The analysis of DNA damage caused by exposure to UV is a major area of research and diagnostics that uses the comet assay [48–50]. Standard protocols and reference materials that could improve the accuracy of comet assay measurements for this and other applications are highly desirable [29, 48, 51].

For better accuracy and minimum variability of the comet assay measurement, the upper limits of the UVA dose and BaP concentration should be about 30 min at an intensity of 2.5 mW/cm^2^, for a dose of 6.3 J/cm^2^ and 10^−6^ mol/L BaP. This would allow adequate cell numbers and accurate measurement of the percentage of DNA in the tail in the comet assay. Also, this level of damaging treatment is near the maximum range of our comet assay measurements, which exhibit almost 100% DNA in the tail, even though the cells have only lost about 20% of their viability. The use of an intermediate UVA treatment level of 2.1 J/cm^2^ (10 min) is well within this range and would be more appropriate for production of a reference material. Preparation of a cellular reference material with this lower loss of viability (5%) would also have wider usage in assay methods, such as the gamma H2AX assay, that rely on the functional DNA damage response of viable cells [52].

A cellular reference material for DNA damage in mammalian cells would need to be produced in substantially large batches for customer distribution. This could be done efficiently using FreeStyle CHO cells, which are derived from the CHO-S cell line and adapted to suspension cell culture. The cells would be grown in a dedicated production system that would enable the cells to be homogeneously exposed in solution at various levels of UVA and BaP. In such arrangement, the whole treated cell population could be sampled for analysis, without regard to issues of cell detachment from the plate surface during growth and treatment, which could lead to artifacts in cell viability and DNA damage measurement. The use of suspended cells could alleviate the problem of insufficient cells for comet analysis at high treatment levels. FreeStyle cell preparations would also be well suited for additional characterization methods that require isolation of DNA, such as mass spectrometry [43]. For the preparation of a reference material, the treated cells would also need to be quickly frozen and stored at −150°C to prevent DNA repair before they are used for subsequent certification by the comet and viability assay and any additional measurements [43]. Certification by multiple methods of comet analysis such as %tail DNA and OTM would be useful. In addition, follow-up measurements would be needed to test the stability of preparations after storage.

## 4. Conclusions

In this study, we have shown that exposure of mammalian cells to increasing levels of UVA dose and BaP concentration has a substantial parallel effect on both cell viability and extent of DNA damage. The loss in viability not only reduces the number of comets available for analysis but also specifies a level of treatment beyond which it is more difficult to accurately calculate the percentage of DNA in the tail as required by the comet assay. Despite this limitation, we find that the use of BaP as a photosensitizer with UVA is a useful method to induce DNA damage in cultured mammalian cells and would be useful in the development of methods and reference materials for the study of UV-induced DNA damage.

## Data Availability

The experimental data used to support the findings of this study are included within the article.
